# Advanced Imaging Integration: Multi-Modal Raman Light Sheet Microscopy Combined with Zero-Shot Learning for Denoising and Super-Resolution

**DOI:** 10.3390/s24217083

**Published:** 2024-11-03

**Authors:** Pooja Kumari, Shaun Keck, Emma Sohn, Johann Kern, Matthias Raedle

**Affiliations:** 1 CeMOS Research and Transfer Center, University of Applied Science, 68163 Mannheim, Germany; s.keck@hs-mannheim.de (S.K.); m.raedle@hs-mannheim.de (M.R.); 2 Universitätsklinikum Mannheim, Universität Heidelberg, 68167 Mannheim, Germany; emma.sohn@uni-heidelberg.de (E.S.); johann.kern@medma.uni-heidelberg.de (J.K.)

**Keywords:** raman scattering, rayleigh scattering, zero-shot deconvolution networks, denoising, fluorescence, light sheet, microscopy, spheroid, multimode, hyperspectral, deep learning, super-resolution

## Abstract

This study presents an advanced integration of Multi-modal Raman Light Sheet Microscopy with zero-shot learning-based computational methods to significantly enhance the resolution and analysis of complex three-dimensional biological structures, such as 3D cell cultures and spheroids. The Multi-modal Raman Light Sheet Microscopy system incorporates Rayleigh scattering, Raman scattering, and fluorescence detection, enabling comprehensive, marker-free imaging of cellular architecture. These diverse modalities offer detailed spatial and molecular insights into cellular organization and interactions, critical for applications in biomedical research, drug discovery, and histological studies. To improve image quality without altering or introducing new biological information, we apply Zero-Shot Deconvolution Networks (ZS-DeconvNet), a deep-learning-based method that enhances resolution in an unsupervised manner. ZS-DeconvNet significantly refines image clarity and sharpness across multiple microscopy modalities without requiring large, labeled datasets, or introducing artifacts. By combining the strengths of multi-modal light sheet microscopy and ZS-DeconvNet, we achieve improved visualization of subcellular structures, offering clearer and more detailed representations of existing data. This approach holds significant potential for advancing high-resolution imaging in biomedical research and other related fields.

## 1. Introduction

**Figure 1:**
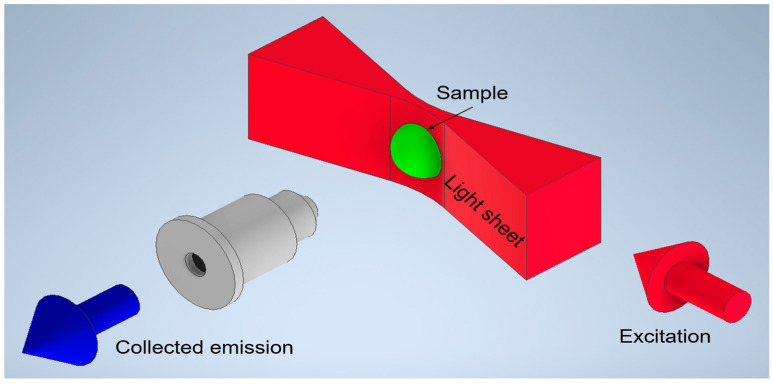
Principle of light sheet microscopy. Excitation and collection axes are orthogonally oriented with the sample placed at their intersection. A laser beam is shaped into a sheet and illuminates a thin section of the sample in the focal plane of the detection objective. The objective images the plane onto a camera chip [2].

Advances in imaging technologies have transformed the study of complex biological systems, particularly in the analysis of three-dimensional (3D) cellular structures. In biomedical research and histology, the ability to accurately visualize and analyze 3D cell cultures, such as spheroids, is critical for understanding cellular behavior, interaction, and function [1]. High-resolution imaging tools and techniques are vital for gaining insights into cellular organization and molecular dynamics, which are essential for fields like drug development and disease modeling. Multi-modal Raman Light Sheet Microscopy has emerged as a highly effective tool for these purposes, combining elastic and inelastic light scattering, including Rayleigh and Stokes Raman scattering, along with fluorescence detection, to provide high-resolution, marker-free imaging of biological samples. The principle of Multi-modal Raman Light Sheet Microscopy is shown in Figure 1. This technique facilitates the reconstruction of comprehensive 3D images, capturing both spatial and molecular information crucial for studies in tissue engineering, cancer biology, and drug development [2,3]. The Multi-modal Raman Light Sheet Microscope is specifically designed to overcome some of the key challenges in biological imaging, such as maintaining the native state of live tissues and cell cultures during imaging [4]. By utilizing the intrinsic molecular properties of Rayleigh and Raman scattering, this technique eliminates the need for external fluorescent markers, thereby reducing potential sample perturbations and preserving physiological conditions [2,5]. This is especially valuable in dynamic, live-cell imaging, where maintaining cellular viability is critical. Moreover, by combining multiple imaging modalities, this system offers a detailed, multi-layered view of both structural and biochemical aspects of the sample, making it a versatile tool in a variety of biomedical applications [6].

However, while Multi-modal Raman Light Sheet Microscopy offers significant advancements in imaging 3D structures, its resolution is still constrained by the diffraction limit of light, and conventional imaging methods are often hampered by noise and signal degradation, particularly in low-light or long-term imaging conditions [2]. To address these challenges, computational super-resolution techniques [7,8] have been developed, with recent breakthroughs in machine learning offering new avenues for enhancing imaging performance [7,9]. Among these, Zero-Shot Deconvolution Networks (ZS-DeconvNet) have shown considerable promise in improving image resolution in real-time and in an unsupervised manner, without the need for large training datasets [10]. ZS-DeconvNet utilizes a CNN-based encoder–decoder structure to achieve computational super-resolution, enhancing spatial resolution by denoising and recovering high-frequency details beyond the optical limits of traditional microscopy. This approach allows visualization of sub-diffraction structures without additional hardware, thereby significantly increasing imaging detail and accuracy. ZS-DeconvNet enhances the resolution of microscope images without requiring ground truth data or additional data acquisition steps, making it particularly suitable for imaging dynamic biological processes [10].

In this study, we incorporate ZS-DeconvNet into multi-modal Raman light sheet microscopy to create a highly advanced imaging platform capable of delivering high-resolution images. The novelty of this approach lies in ZS-DeconvNet’s zero-shot learning capability, allowing adaptive image enhancement across multiple microscopy modalities without pre-training or modality-specific tuning. This multimodal adaptability provides a unified solution for image enhancement, efficiently overcoming modality-specific challenges in fluorescence, Raman, and other microscopy techniques. By integrating cutting-edge computational techniques with Multi-modal Light Sheet Microscopy, we aim to significantly improve both the spatial and molecular resolution of biological imaging [11,12,13]. This combined approach provides new opportunities for real-time visualization of complex cellular structures and dynamic processes, with far-reaching implications for biomedical research, cellular biology, and therapeutic development. Spheroids derived from head and neck squamous cell carcinoma (HNSCC) are particularly valuable in cancer research due to their resemblance to in vivo tumor architecture and behavior, including response to chemotherapeutics. In this study, we use UMSCC-11B cells, which are derived from HPV-negative HNSCC cell lines, to demonstrate the capabilities of Multi-modal Raman Light Sheet Microscopy enhanced by ZS-DeconvNet. Additionally, we explore the effect of the chemotherapeutic agent cisplatin on these spheroids, providing insights into both imaging and drug-response dynamics.

## 2. Materials and Methods

### 2.1. The Enhanced Multi-Modal Raman Light Sheet Microscopy with Zero-Shot Denoising Integration

This study utilizes an advanced Multi-modal Raman Light Sheet Microscope combining Rayleigh scattering, Raman scattering, and fluorescence emission for high-resolution, two-dimensional imaging of biological samples such as 3D spheroids and cell cultures [2,14,15,16]. The system integrates Zero-Shot Deconvolution Networks (ZS-DeconvNet), an advanced unsupervised deep learning technique to enhance image quality by significantly reducing noise without the need for additional training datasets or reference images [10,17].

### 2.2. Multi-Model Light Sheet Microscope Design

**Figure 2:**
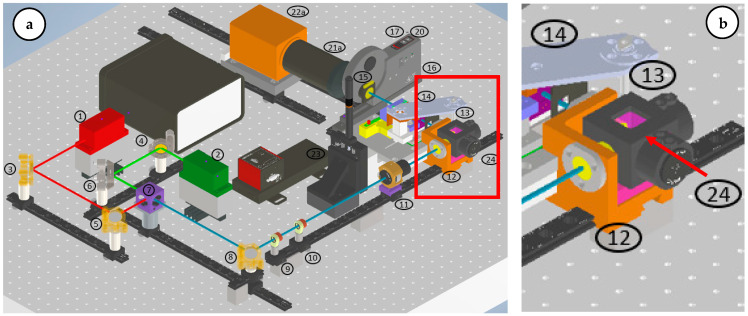
(**a**) Isometric view of the Raman light sheet microscope CAD model with connected sCMOS camera. The colored lines indicate the optical path of the illuminating lasers. Red: 660 nm beam propagation. Green: 785 nm beam propagation. Blue coaxial superimposed 660 nm and 785 nm beam propagation [2]. (**b**) Sample chamber (24), where sample is placed.

The Multi-modal Raman Light Sheet Microscope is based on the OpenSPIM platform [18], with significant enhancements to incorporate multi-modal imaging capabilities. The optical system consists of three primary components: the beam-shaping and illumination optics, the spectral selection and imaging optics, and the precision sample positioning system (Figure 2) (Table 1).

- Excitation Lasers: Two continuous-wave lasers, with emission wavelengths of 660 nm and 785 nm, are used to excite Rayleigh and Raman scattering, respectively. The 660 nm laser is optimized for fluorescence imaging while minimizing autofluorescence, and the 785 nm laser enhances Raman scattering signals. Adjustable power outputs (0.5–130 mW for 660 nm and 0.5–200 mW for 785 nm) allow fine control of illumination intensity. Both laser beams are collimated and aligned coaxially using a series of broadband mirrors and dichroic splitters.
- Beam Shaping: The laser beams are expanded using a Keplerian telescope system formed by achromatic doublets, which increase the illuminated field of view without compromising beam focus. A cylindrical lens focuses the expanded beam into a static light sheet, projected into the sample chamber through a 10× water immersion objective.
- Imaging Optics: Photons scattered and emitted by the sample are collected by a 20× water immersion detection objective, positioned orthogonally to the light sheet for optimal detection. An Acousto-Optic Tunable Filter (AOTF) enables precise spectral selection with a 2 nm bandwidth, allowing for fine control over the wavelengths collected. Additional long-pass and short-pass filters further refine the detected signal. A high-sensitivity sCMOS camera (Hamamatsu ORCA Flash 4.0 LT+) is used for image acquisition, operating in a 1024 × 1024 pixel mode optimized for low-light conditions.

### 2.3. Sample Preparation and Positioning

Biological samples, including 3D spheroids and cell cultures, were prepared following standard protocols. Samples were embedded in a low-scattering hydrogel matrix, ensuring both optical transparency and the preservation of physiological conditions during imaging. This approach minimized scattering while maintaining an environment conducive to cellular function.

#### 2.3.1. Cell Culture and Spheroid Formation

For this study, spheroids were generated using HPV-negative head and neck squamous cell carcinoma (HNSCC) cell lines, UMSCC-11B, provided by Dr. Thomas Carey from the University of Michigan. UMSCC-11B was derived from a laryngeal carcinoma.

**Monoculture Spheroids**

The UMSCC-11B cell lines were cultured in Eagle’s Minimum Essential Medium (EMEM, Lonza, United States (Walkersville, Maryland)), supplemented with 10% fetal bovine serum (FBS) and 1% Penicillin/Streptomycin (Pen/Strep). Cells were incubated at 37 °C in a humidified atmosphere with 5% CO_2_. Once cells reached confluency, they were washed with Dulbecco’s phosphate-buffered saline (DPBS) and detached using Trypsin/EDTA. The total number of cells was determined using a Neubauer hemocytometer.

For spheroid formation, UMSCC-11B cells were seeded into 96-well ultra-low attachment (ULA) round-bottom plates (ThermoFisher Scientific, Mannheim, Germany) at a density of 2.5 × 10^4^ or 5 × 10^4^ cells per well. Spheroids were cultured for up to eight days, with media changes on days 3, 5, and 8. After spheroids reached the desired size, typically around 300–400 µm in diameter, they were prepared for subsequent imaging experiments.

**Drug Treatment of Spheroids**

To explore the effects of drug treatment, spheroids were treated with the chemotherapy drug cisplatin (Selleck Chemicals) during the course of their formation. On day 4 of spheroid culture, cisplatin was added to designated wells at concentrations of 50 µM or 100 µM, while control spheroids were treated with an equivalent volume of dimethyl sulfoxide (DMSO). Following drug treatment, the spheroids were incubated for an additional 48 or 72 h to assess the impact on morphology and viability.

**Spheroid Fixation**

At the end of the treatment period, both treated and untreated spheroids were fixed in 4% formalin for subsequent imaging using multi-modal Raman light sheet microscopy. Fixation ensured the structural integrity of the spheroids during the imaging and analysis processes.

#### 2.3.2. Sample Mounting

**Figure 3:**
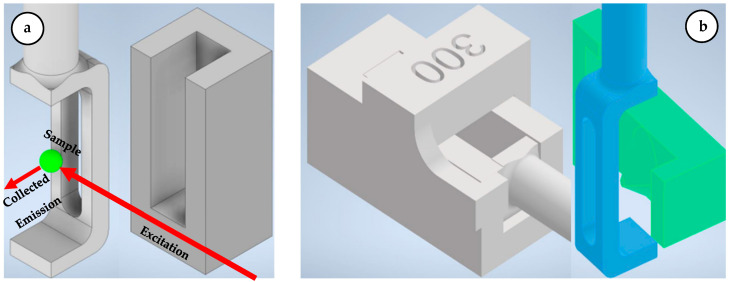
(**a**) CAD model of a multi-view sample carrier and corresponding frame for embedding spheroid samples in hydrogels. This sample is located in the sample chamber shown in Figure 2b. (**b**) Sample holder system consisting of gel chamber with cylindrical extension, casting frame and negative mold for precise, reproducible embedding of spheroids in hydrogel.

For imaging, the spheroids were mounted in a custom-designed 3D-printed hydrogel carrier (Figure 3a,b). This carrier was optimized to precisely align the spheroids within the light sheet and the focal plane of the detection objective. Additionally, the carrier’s design allowed for rotational adjustments, facilitating multi-view imaging from different angles.

#### 2.3.3. Environmental Control

During imaging, the sample chamber was maintained at 37 °C, with a regulated atmosphere containing 5% CO_2_ to preserve sample viability over the course of extended imaging sessions. A 4D positioning stage enabled precise movement along the X, Y, and Z axes, with rotational adjustments for consistent sample alignment and accurate positioning within the microscope’s field of view.

### 2.4. Zero-Shot Deconvolution Network (ZS-DeconvNet)

ZS-DeconvNet is an advanced machine-learning-based denoising algorithm designed to enhance image quality by reducing noise and preserving fine structural details. Unlike traditional supervised deep learning methods, ZS-DeconvNet operates in an unsupervised manner, requiring no ground-truth data or pre-trained models [10,19].

**Mathematical Model of ZS-DeconvNet**

The network minimizes noise in the acquired images using a self-supervised learning framework. The objective function for ZS-DeconvNet is formulated as:

$$L\left(\theta \right)={\left|\left|I_{raw}-I_{denoised}\left(\theta \right)\right|\right|}^{2}+\lambda |\left|\nabla I_{denoised}\left(\theta \right)\right||$$

where:

$I_{raw}$ represents the noisy input image.

$I_{denoised}\left(\theta \right)$ represents the output of the neural network after applying the network’s parameters $\left(\theta \right)$.

λ is a regularization term controlling the smoothness of the denoised image.

$\nabla I_{denoised}\left(\theta \right)$ represents the image gradients, ensuring that edges are preserved during denoising.

The Zero-Shot Deconvolution Network (ZS-DeconvNet) was implemented to improve the quality of the Raman Light Sheet Microscopy images by reducing noise and enhancing resolution.

**Network Architecture: ZS-DeconvNet**

The ZS-DeconvNet is built on a CNN-based encoder-decoder architecture. The encoder compresses the input image into a lower-dimensional latent space through a sequence of Conv2D layers, each followed by batch normalization and max pooling. These layers are designed to extract critical structural features from the image while progressively reducing its dimensionality, enabling the model to focus on the most important information. The decoder mirrors this process, gradually reconstructing the image by applying upsampling and Conv2D layers. Additionally, skip connections between the encoder and decoder allow the model to retain high-resolution details by concatenating features from earlier layers. Finally, a sigmoid-activated Conv2D layer produces the denoised output.

**Figure 4:**
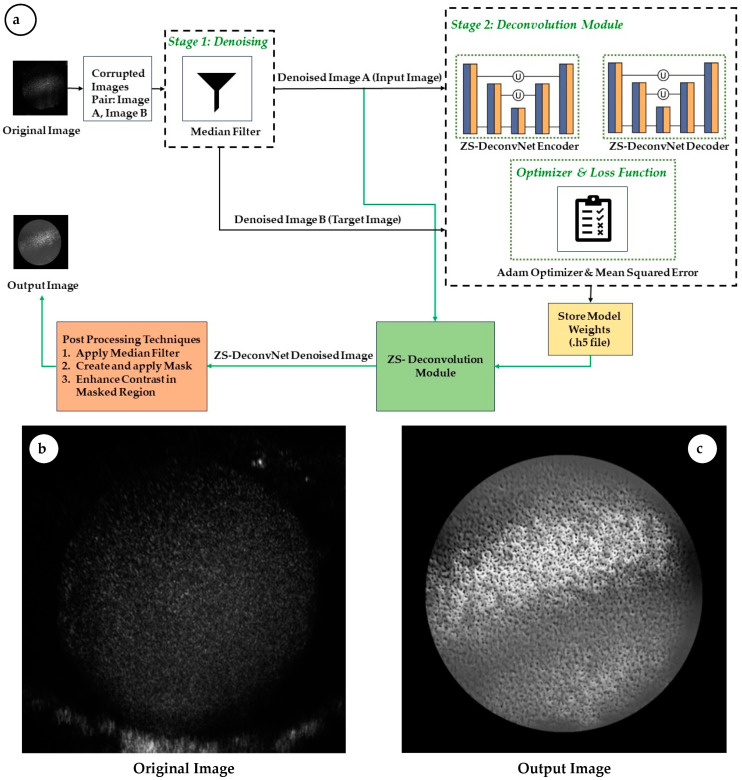
(**a**) The Zero-Shot Deconvolution Network (ZS-DeconvNet) architecture outlines the training workflow, encompassing pre-processing steps—such as corrupted image generation and median filter-based denoising—as well as post-processing techniques, including region-of-interest (ROI) image enhancement and morphological operations. The network’s performance is assessed using PSNR, SSIM, and RMSE metrics to achieve enhanced image quality in Raman light sheet microscopy. (**b**,**c**) represent the input (**b**) and output (**c**) of the ZS-DeconvNet architecture, as depicted in (**a**).

During training, ZS-DeconvNet follows a zero-shot learning approach. Two corrupted versions of the same image are generated—Denoised Image A and Denoised Image B (Figure 4)—by adding and subtracting noise, respectively. The model learns to map Denoised Image A (input) to Denoised Image B (target) without requiring a clean reference image. This is achieved using a Mean Squared Error (MSE) loss function, which minimizes the pixel-wise difference between the predicted and target images. By optimizing the MSE, the network progressively improves its ability to remove noise and restore details from noisy input data. Once the ZS-DeconvNet model is trained, it can be applied to new, unseen noisy images, resulting a denoised version of the input image by leveraging the learned features from the training process(Table 2).

The ZS-DeconvNet is designed to generalize well to unseen data. The lack of a need for pre-trained datasets enhances its flexibility, allowing it to be applied in image enhancement tasks. This feature is particularly advantageous in applications such as cell imaging and video microscopy, where pre-trained datasets may not be available or applicable (Figure 4).

**Performance Evaluation Metrics**

To rigorously assess the performance of the ZS-DeconNet model in image enhancement, we calculated various performance metrics: Peak Signal-to-Noise Ratio (PSNR), Structural Similarity Index (SSIM), Root Mean Square Error and Fourier Ring Correlation (FRC). PSNR is used to assess the fidelity of a processed image in relation to an uncorrupted reference. Higher PSNR values suggest that the denoised image retains fidelity to the original structural and intensity details, confirming effective noise reduction and minimal loss of crucial information. PSNR thus serves as a direct indicator of ZS-DeconvNet’s ability to faithfully restore high-resolution detail, which is essential for accurate imaging in microscopy. SSIM is a perceptual metric that quantifies structural similarity by evaluating luminance, contrast, and spatial composition between the original and processed images. Elevated SSIM scores demonstrate that ZS-DeconvNet not only reduces noise but also maintains the image’s inherent structural relationships, essential for preserving context in microscopy where spatial coherence is key to interpretation. SSIM is especially beneficial for validating that enhanced images accurately reflect the morphology and structural integrity of biological samples. RMSE quantifies the average deviation in pixel intensity between the denoised and original images, offering a robust measure of reconstruction accuracy. Lower RMSE values denote minimal divergence from the expected pixel values, highlighting the model’s ability to precisely restore image content even under significant noise interference. To objectively evaluate improvements in spatial resolution, we utilize Fourier Ring Correlation (FRC), a frequency-domain metric that quantitatively assesses resolution by comparing spatial frequency content before and after processing. FRC is widely used in super-resolution microscopy and quantifies the effective resolution enhancement achieved by ZS-DeconvNet. Unlike PSNR, SSIM, and RMSE, which primarily address image quality and similarity, FRC directly measures resolution improvements, providing insight into the model’s ability to recover or even enhance fine structural details. By achieving a higher FRC resolution threshold, ZS-DeconvNet confirms its utility in super-resolution applications, effectively distinguishing it as a powerful tool for high-resolution image enhancement in multimodal microscopy.

Pre-processing: Before being fed into the ZS-DeconvNet, the raw images (Original Image) undergo a series of crucial preprocessing steps aimed at preparing the data for optimal denoising and model training. In the initial step I, two corrupted image pairs (Image A, Image B) were generated by introducing Gaussian noise to the original image, where Image A had added noise and Image B had inverted noise. After creating a corrupted image pair from the Original Image, median filtering was applied to these images to remove high-intensity “salt-and-pepper” noise, which is commonly seen in images captured through noisy channels, such as biomedical imaging. A median filter with a kernel size of 3 was applied, effectively suppressing noise while preserving edges and fine details within the image. This enhances the network’s ability to focus on meaningful structural elements during training and inference, contributing to more precise noise removal. (ref. Figure 4)

These pairs serve as input (Denoised Image A) and target (Denoised Image B) images during the training phase of ZS-DeconvNet. The model learns to predict Denoised Image B from Denoised Image A, simulating a noise-to-noise learning framework.

Post-processing: Following the ZS-DeconvNet denoising process, additional post-processing steps were performed, such as applying a region of interest (ROI) mask, applying Median Filter with Kernal size 3, and enhancing contrast to refine the output image. Here we also calculated PSNR, SSIM, RMSE, and FRC metrics for performance evaluation [19,20]. These enhancements further improved the clarity and usability of the denoised image by focusing on key structures (Figure 4).

These operations help in emphasizing small structures within the spheroids, particularly in the cellular boundaries. If required, edge detection algorithms (such as Canny edge detection) can also be applied to highlight critical boundaries and structural details. This step is essential in the analysis of biological images, where the accurate delineation of subcellular components plays a crucial role in data interpretation.

## 3. Results

### 3.1. Denoising Performance and Image Clarity

This study assesses the denoising performance of ZS-DeconvNet on 11B spheroid samples, comparing both treated and untreated conditions following exposure to 50 µM cisplatin for 72 h (refer to the Sample Preparation section for further details). Images were captured using two laser excitations, 660 nm and 785 nm, and processed across multiple imaging modalities. The primary objective was to determine the effectiveness of ZS-DeconvNet in reducing noise while preserving critical image features, and to identify any structural or molecular changes induced by the treatment in the spheroids.

**Figure 5:**
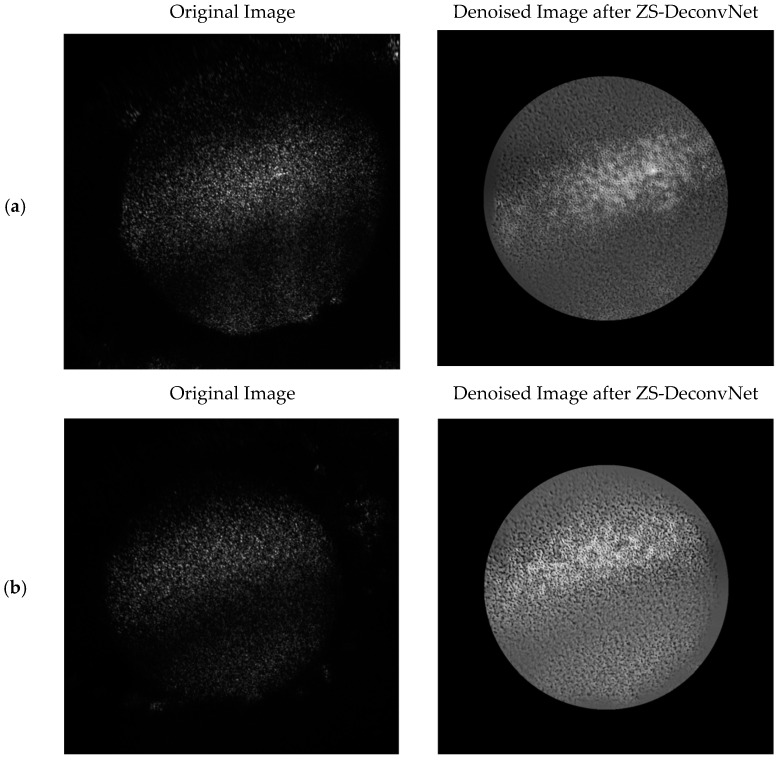
Comparison of original 11B-Untreated (**a**) and 11B-Treated Cells (**b**) images and denoised images after ZS-DeconvNet obtained using laser excitation at 660 nm and AOTF at 650 nm (Rayleigh scattering).

**Figure 6:**
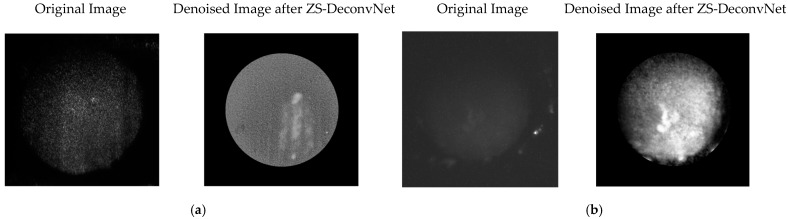
Comparison of original 11B-Untreated Cells (**a**) and 11B-Treated Cells (**b**) images and denoised images after ZS-DeconvNet obtained using laser excitation at 660 nm and AOTF at 817 nm (Raman scattering).

**Figure 7:**
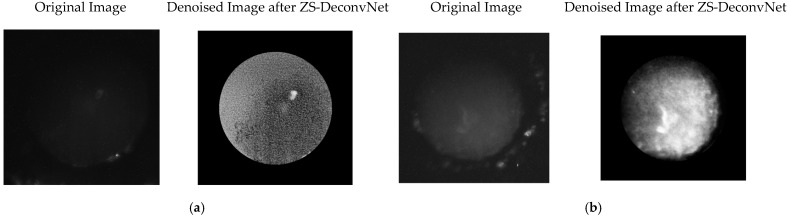
Comparison of original 11B-Untreated Cells (**a**) and 11B-Treated Cells (**b**) images and denoised images after ZS-DeconvNet obtained using laser excitation at 660 nm and AOTF at 694 nm (fluorescence).

Laser Excitation at 660 nm: For the 660 nm laser, three distinct modalities were used:

(1) Rayleigh Scattering (Power: 1 mW and AOTF: 650 nm): The raw images captured using 660 nm Rayleigh scattering were heavily impacted by noise, making it difficult to discern fine structural details in both treated and untreated spheroids. As demonstrated in Figure 5a,b, the original image (left) contains substantial noise that obscures surface-level information. After applying ZS-DeconvNet, the denoised image (right) exhibited a marked reduction in noise, allowing for the visualization of key features that were previously hidden. The treated spheroids exposed to 50 µM cisplatin for 72 h revealed subtle structural alterations, such as surface roughness and changes in texture, which were not discernible in the noisy image. ZS-DeconvNet’s ability to enhance image clarity at such low power (1 mW) demonstrates its robustness in handling noisy datasets without sacrificing the essential information within the image.

(2) Raman Scattering (Power: 130 mW and AOTF: 817 nm): Denoising significantly enhanced the signal-to-noise ratio (SNR), enabling clearer identification of molecular changes induced by 50 µM cisplatin. Treated spheroids showed distinct Raman shifts and enhanced peaks, while untreated spheroids maintained stable profiles. ZS-DeconvNet preserved these features, improving interpretability (Figure 6).

(3) Fluorescence (Power: 130 mW, AOTF: 694 nm): Fluorescence imaging showed substantial improvement after denoising, with noise suppression enhancing signal clarity. Treated spheroids exhibited increased fluorescence intensity, indicating structural or cellular changes, while untreated spheroids displayed more uniform fluorescence. ZS-DeconvNet preserved signal integrity, making the fluorescence data more interpretable (Figure 7).

**Figure 8:**
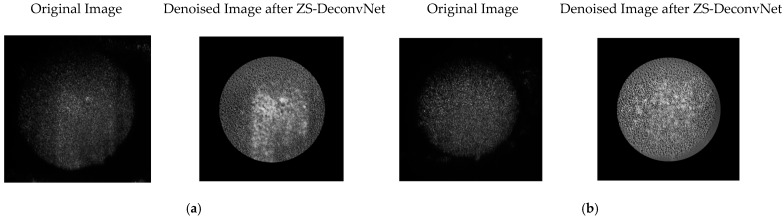
Comparison of original 11B-Untreated Cells (**a**) and 11B-Treated Cells (**b**) images and denoised images after ZS-DeconvNet obtained using laser excitation at 785 nm and AOTF at 775 nm (Rayleigh scattering).

Laser Excitation at 785 nm: For the 775 nm laser, three distinct modalities were used:

(1) Rayleigh Scattering (Power: 1 mW and AOTF: 775 nm): In the 785 nm Rayleigh scattering modality, ZS-DeconvNet provided substantial image quality enhancement. The denoised images of cisplatin-treated spheroids revealed previously masked surface irregularities, such as increased roughness and textural changes, that were critical for assessing treatment effects (Figure 8).

### 3.2. Quantitative Evaluation of Image Quality

**Figure 9:**
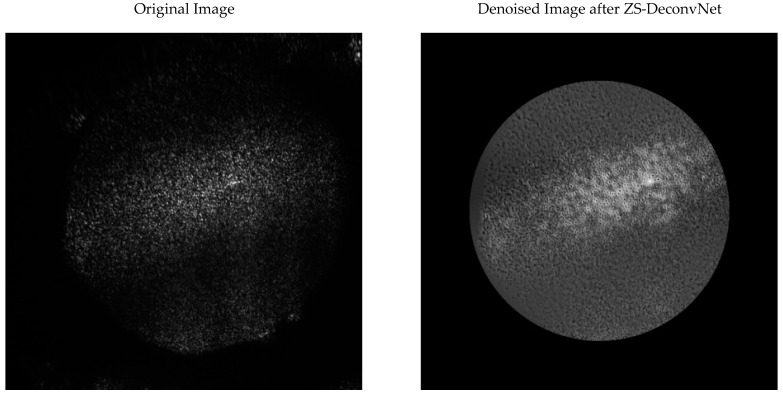

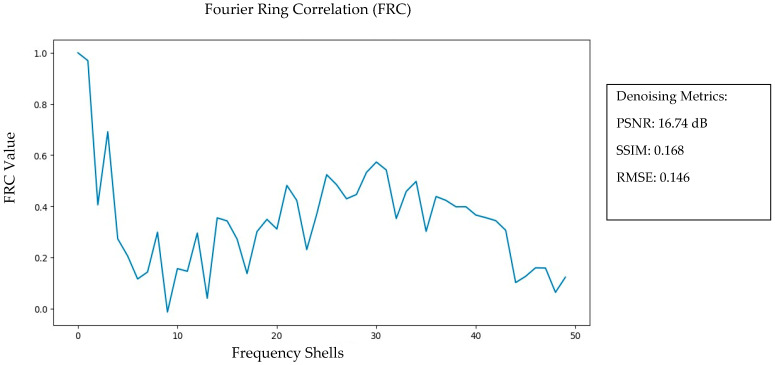
Denoising performance of ZS-DeconvNet on 11B-Untreated cells images using 660 nm laser for Rayleigh spectra: original image, denoised image after ZS-DeconvNet, FRC analysis and denoising metrics (PSNR, SSIM and RMSE).

The denoising capabilities of ZS-DeconvNet were quantitatively assessed using Peak Signal-to-Noise Ratio (PSNR), Structural Similarity Index (SSIM), and Root Mean Square Error (RMSE), as shown in Figure 9. These metrics provide a comprehensive evaluation of how well the denoised images preserve structural integrity and reduce noise, enhancing the overall quality of the data for further analysis.

PSNR: The denoised image achieved a PSNR value of 16.74 dB, indicating a substantial reduction in noise when compared to the raw image. Although this PSNR value reflects moderate image fidelity, it represents a significant improvement in signal clarity, allowing for better visualization of features previously masked by noise. This improvement underscores the effectiveness of ZS-DeconvNet in restoring image quality in high-noise conditions such as Rayleigh scattering at 660 nm.

SSIM: The SSIM score of 0.168 indicates that some structural information was preserved post-denoising, although there is room for further optimization. Despite the relatively low score, this increase in structural similarity highlights ZS-DeconvNet’s ability to recover key features from the noisy input, enabling a more interpretable output. This is particularly relevant in imaging modalities where fine structural details are critical for accurate analysis, such as in the treated spheroids exposed to cisplatin.

RMSE: The RMSE value of 0.146 demonstrates the model’s efficiency in minimizing the error between the original noisy image and the denoised output. The reduction in RMSE confirms that ZS-DeconvNet effectively suppresses noise without introducing artifacts, thereby preserving essential structural and molecular details crucial for the accurate evaluation of treatment effects in cisplatin-treated spheroids.

In addition to these metrics, Fourier Ring Correlation (FRC) analysis (Figure 1) was conducted to further evaluate the resolution enhancement. The FRC curve shows improved frequency preservation across multiple shells, indicating that ZS-DeconvNet enhanced the image’s spatial resolution while maintaining relevant frequency details. This result highlights the model’s capability to improve image quality even in noisy and low-signal environments.

Overall, these quantitative metrics affirm the robustness of ZS-DeconvNet in effectively denoising images, particularly in the context of high-noise biomedical imaging applications, facilitating clearer feature extraction and more reliable data interpretation for both treated and untreated conditions.

## 4. Discussion

This study highlights the effectiveness of ZS-DeconvNet in combination with Multi-modal Raman Light Sheet Microscopy for high-resolution imaging of spheroids derived from UMSCC-11B cell lines. The ability to visualize both structural and molecular changes in spheroids exposed to 50 µM cisplatin for 72 h significantly advanced our understanding of treatment-induced effects. By leveraging the denoising capabilities of ZS-DeconvNet, we were able to enhance the clarity of images across multiple imaging modalities, with a particular emphasis on Raman scattering channels [2,10].

Noise Reduction and Image Clarity: The major finding of this work is the significant improvement in signal-to-noise ratio (SNR) achieved by ZS-DeconvNet, which enabled the extraction of valuable structural and molecular details that were otherwise obscured by noise. This was particularly evident in the 660 nm Rayleigh scattering and Raman scattering channels, where noise levels are typically high due to the sensitivity of these modalities to low signal intensities. The PSNR improvements in denoised images reflect the model’s ability to suppress noise without compromising the integrity of critical image features. Additionally, FRC analysis confirmed that the high-frequency components of the images were well-preserved, leading to better resolution and sharper image details, which is essential for studying subtle structural changes in spheroids. The ability to reveal treatment-induced surface irregularities and molecular shifts in cisplatin-treated spheroids demonstrates the utility of ZS-DeconvNet in high-noise imaging environments. For instance, Raman scattering at 660 nm (130 mW, AOTF: 817 nm), which is particularly sensitive to molecular vibrations, revealed distinct shifts in the spectral profiles of treated spheroids after denoising. These shifts were crucial for identifying treatment-induced molecular changes, which were previously masked by noise in the raw images. In contrast, the untreated spheroids maintained stable Raman profiles, further emphasizing the specificity of the cisplatin-induced changes and the model’s effectiveness in differentiating between treated and control conditions.

Structural Preservation and Molecular Insights: While ZS-DeconvNet excelled in noise reduction, as evidenced by improved PSNR and RMSE values, the SSIM scores indicate that there is room for further optimization, particularly in preserving intricate structural details. Nevertheless, the overall structural integrity of the denoised images was maintained, as demonstrated by the clear visualization of cisplatin-induced surface irregularities in treated spheroids. This preservation of structural features is critical in biomedical imaging, where even slight distortions can lead to misinterpretation of biological changes. The ability to retain structural fidelity while reducing noise enabled the detection of molecular changes that provide deeper insights into the spheroids’ responses to cisplatin treatment. Fluorescence imaging at 660 nm (130 mW, AOTF: 694 nm) benefitted significantly from denoising, with the treated spheroids displaying increased fluorescence intensity, suggesting possible alterations in cell viability or metabolic activity. The preserved fluorescence signals in denoised images allowed for more accurate assessments of these biological processes, facilitating a deeper understanding of treatment-induced cellular changes.

Implications for Biomedical Research: The application of ZS-DeconvNet in this study offers substantial implications for biomedical research, particularly in fields such as cancer biology, drug discovery, and tissue engineering. The ability to visualize real-time molecular changes in 3D spheroids, which are physiologically relevant models for tumor behavior, provides critical insights into how treatments like cisplatin affect cellular architecture and molecular composition. Furthermore, the flexibility of ZS-DeconvNet—which does not require extensive pre-training on specific datasets—makes it a versatile tool for various imaging modalities and experimental setups. Additionally, the integration of post-processing techniques such as image segmentation, contrast enhancement and edge detection can further enhance the usability of the denoised images for downstream analysis. These enhancements ensured that the images were ready for detailed analysis, such as subcellular structural studies or quantitative assessments of spheroid viability. The results of this study suggest that ZS-DeconvNet, when combined with advanced imaging modalities, can significantly improve the quality of data available for quantitative biomedical research.

Future Directions: While ZS-DeconvNet demonstrated strong denoising performance, future research could explore hybrid approaches that integrate the noise suppression capabilities of ZS-DeconvNet with advanced structural preservation techniques. This would ensure even higher SSIM values while maintaining the improvements in PSNR and RMSE. Furthermore, integrating ZS-DeconvNet with deep learning-based segmentation techniques could open new avenues for automated analysis of spheroid morphology and molecular dynamics in response to various treatments. In conclusion, this study demonstrates that ZS-DeconvNet, combined with Multi-modal Raman Light Sheet Microscopy, offers a powerful and flexible framework for imaging 3D spheroids. The model’s ability to denoise images in real-time without sacrificing critical structural or molecular information makes it an invaluable tool for biomedical research. By providing high-quality, denoised images that are ready for detailed analysis, ZS-DeconvNet facilitates a more precise understanding of treatment effects on live cells and tissues, paving the way for new applications in drug discovery, cancer research, and tissue engineering.

## 5. Conclusions

This study evaluated the performance of ZS-DeconvNet for denoising high-noise biomedical images of 11B spheroids treated with 50 µM cisplatin for 72 h. Metrics like PSNR, SSIM, RMSE, and FRC demonstrated the model’s ability to significantly reduce noise while preserving important structural and molecular details.

The model showed a marked improvement in PSNR, confirming its effectiveness in noise suppression and image clarity, while FRC analysis highlighted its ability to retain high-frequency information. This approach enables resolution enhancement beyond the diffraction limit by recovering high-frequency details, allowing for visualization of sub-diffraction structures without additional hardware adjustments. Although SSIM scores indicated some limitations in preserving fine details, ZS-DeconvNet successfully maintained key features, especially in Rayleigh and Raman scattering modalities at both 660 nm and 785 nm. Its zero-shot learning framework further underscores the novelty of this multimodal approach, allowing adaptive enhancement across multiple imaging modalities without the need for pre-trained models. This flexibility addresses unique challenges of multimodal microscopy, providing a unified solution for image enhancement in Raman, fluorescence, and other microscopy techniques.

Denoised images revealed critical treatment-induced changes in cisplatin-treated spheroids, previously masked by noise, enabling more accurate comparisons between treated and untreated samples. This underscores ZS-DeconvNet’s effectiveness in high-noise, low-signal imaging environments typical of biomedical applications.

Overall, ZS-DeconvNet provides a powerful tool for real-time image denoising in 3D spheroid imaging and Raman Light Sheet Microscopy, offering faster processing and superior image quality without needing pre-trained datasets. Future research could focus on hybrid approaches to combine its noise reduction capabilities with advanced structural preservation techniques for even better results in biomedical imaging.

## Figures and Tables

**Table 1:** Optical and mechanical components used in the Raman light sheet microscope.

No.	Component Specification	Manufaturer
1	LuxX Laser 785 nm, adjustable laser power 0.5–200 mW	Omicron GmbH (Dudenhofen Germany)
2	LuxX Laser 660 nm, adjustable laser power 0.5–130 mW	Omicron GmbH
3,5,8	Broadband mirror, Ø25.4 mm, EO2 coated, mounted in Polaris K1 Kinematic Mirror Mount	Thorlabs GmbH (Lübeck, Germany)
4,6	Broadband mirror, Ø25.4 mm, EO3 coated, mounted in Polaris K1 Kinematic Mirror Mount	Thorlabs GmbH
7	BrightLine laser dichroic beamsplitter, 25 mm × 36 mm, reflection band 350–671 nm, transmission band 702–1200 nm	Semrock (New York, NY, USA)
9	Mounted achromatic doublet lens, Ø12.7 mm, focal length 25 mm, anti-reflex coating 400–1100 nm	Thorlabs GmbH
10	Mounted achromatic doublet lens, Ø12.7 mm, focal length 50 mm, anti-reflex coating 400–1100 nm	Thorlabs GmbH
11	Mounted cylindrical achromatic doublet lens, Ø25.4 mm, focal length 50 mm, anti-reflex coating 650–1050 nm	Thorlabs GmbH
12	UMPLFLN10XW water dipping objective, magnification 10×, numerical aperture 0.3, working distance 3.5 mm	Evident (Hamburg, Germany)
13	UMPLFLN20XW water dipping objective, magnification 20×, numerical aperture 0.5, working distance 3.5 mm	Evident
14	Acousto-Optic Tunable Filter (AOTF), spectral range 550–1000 nm	Brimrose
15	Polarization filter	Thorlabs GmbH
16	6-position motorized filter wheel	Thorlabs GmbH
17	Longpass filter, 660 nm	Semrock
18	Notch filter, 660 nm	Semrock
19	Shortpass filter, 660 nm	Semrock
20	Longpass filter, 785 nm	Semrock
21a	Tube lens U-TLU and C-mount (U-TV0.5XC-3)	Evident
21b	Aspheric condenser lens, Ø25 mm, focal length 20 mm, anti-reflex coating 650–1050 nm	Thorlabs GmbH
22a	sCMOS camera ORCA Flash 4.0 LT+	Hamamatsu (Herrsching, Germany)
22b	CXY1 two-axis translating lens mount, Ø550 µm optic fiber	Thorlabs GmbH
23	USB-4D stage (X, Y, Z, R)	Picard-Industries (Albion, NY, USA)
24	Sample chamber, aluminum mounting frame, acrylic water chamber	CeMOS Research and Transfer Center (Mannheim, Germany)
25	MultiSpec^®^ Raman spectrometer	tec5 GmbH (Steinbach, Germany)

**Table 2:** Description of Parameters/Hyperparameters used during ZS-DeconvNet Training.

ZS-DeconvNet Parameters/Hyperparameters	Description/Details
Input Image type	tiff/.tif
Input Image Size	1024 × 1024
Loss Function	Mean Squared Error
Optimizer	Adam
Epochs	100
Batch Size	1
Learning Rate	0.001
Evaluation Metrics	PSNR, SSIM, RMSE, FRC

## Data Availability

Data are contained within the article.
